# Vaginal innate immune mediators are modulated by a water extract of *Houttuynia cordata* Thunb

**DOI:** 10.1186/s12906-015-0701-9

**Published:** 2015-06-16

**Authors:** Surada Satthakarn, Florian Hladik, Aornrutai Promsong, Wipawee Nittayananta

**Affiliations:** Department of Biomedical Sciences, Faculty of Medicine, Prince of Songkla University, Hat Yai, Songkhla 90110 Thailand; Department of Obstetrics and Gynecology, University of Washington, Seattle, WA 98195 USA; Vaccine and Infectious Disease Division, Fred Hutchinson Cancer Research Center, Seattle, WA 98195 USA; Faculty of Medicine, Princess of Naradhiwas University, Narathiwat, 96000 Thailand; Excellent Research Laboratory, Phytomedicine and Pharmaceutical Biotechnology Excellence Center, Hat Yai, Songkhla 90110 Thailand; Natural Product Research Center of Excellence, Faculty of Science, Prince of Songkla University, Hat Yai, Songkhla 90110 Thailand; Graduate School, Prince of Songkla University, Hat Yai, Songkhla 90110 Thailand

**Keywords:** Chemokines, Cytokines, Female reproductive tract, hBD2, Mucosal innate immunity, SLPI, Vaginal epithelial cells

## Abstract

**Background:**

Vaginal epithelial cells (VECs) produce antimicrobial peptides including human β-defensin 2 (hBD2) and secretory leukocyte protease inhibitor (SLPI), as well as cytokines and chemokines that play vital roles in mucosal innate immunity of the female reproductive tract. *Houttuynia cordata* Thunb (*H. cordata*), a herbal plant found in Asia, possesses various activities including antimicrobial activity and anti-inflammation. As inflammation and infection are commonly found in female reproductive tract, we aimed to investigate the effects of *H. cordata* water extract in modulating innate immune factors produced by VECs.

**Methods:**

Primary human VECs were cultured and treated with *H. cordata* at a concentration ranging from 25–200 μg/ml for 6 or 18 h. After treatment, the cells and culture supernatants were harvested. The expression of hBD2 and SLPI mRNA was evaluated by quantitative real-time reverse transcription PCR. Levels of secreted hBD2 and SLPI as well as cytokines and chemokines in the supernatants were measured by ELISA and Luminex assay, respectively. Cytotoxicity of the extract on VECs was assessed by CellTiter-Blue Cell Viability Assay.

**Results:**

*H. cordata* did not cause measurable toxicity on VECs after exposure for 18 h. The expression of hBD2 and SLPI mRNA as well as the secreted hBD2 protein were increased in response to *H. cordata* exposure for 18 h when compared to the untreated controls. However, treatment with the extract for 6 h had only slight effects on the mRNA expression of hBD2 and SLPI. The secretion of IL-2 and IL-6 proteins by VECs was also increased, while the secretion of CCL5 was decreased after treatment with the extract for 18 h. Treatment with *H. cordata* extract had some effects on the secretion of IL-4, IL-8, CCL2, and TNF-α, but not statistically significant.

**Conclusions:**

*H. cordata* water extract modulates the expression of antimicrobial peptides and cytokines produced by VECs, which play an important role in the mucosal innate immunity in the female reproductive tract. Our findings suggest that *H. cordata* may have immunomodulatory effects on the vaginal mucosa. Further studies should be performed *in vivo* to determine if it can enhance mucosal immune defenses against microbial pathogens.

## Background

The lower female reproductive tract is the site of entry for several pathogens including bacteria, yeasts, and viruses that may cause sexually transmitted infections [1, 2]. The immune system of mucosal surfaces functions as the first line of host defense against pathogen invasion to protect underlying tissues and organs [1, 3]. The vaginal epithelium plays an important role in the innate immunity of the female reproductive tract, by providing a physical barrier and producing antimicrobial peptides, such as human β-defensin 2 (hBD2) and secretory leukocyte protease inhibitor (SLPI), and other innate immune mediators, in particular cytokines and chemokines [2, 4–6]. These immune mediators possess potent antimicrobial activities against a broad range of infectious pathogens, including human immunodeficiency virus type 1 (HIV-1) and *Candida albicans*, and play significant roles in host immune responses and homeostasis [7–14].

Previous studies revealed that hBD2 and SLPI can inhibit HIV-1 infection [7, 10, 15, 16]. However, the secretion of hBD2 and SLPI in cervicovaginal lavages may fluctuate during a menstrual cycle [2]. In addition, levels of SLPI protein have been shown to be decreased in the cervicovaginal secretions of post-menopausal women [17]. These may lead to impairment of the immune responses and make those women become susceptible to immune-mediated vaginal infections. Thus, the induction of these immune mediators may help in maintaining mucosal immunity and preventing microbial invasion.

Some of these immune mediators are constitutively produced by vaginal epithelial cells (VECs). They can also be up-regulated by different microbial components and cytokines [4, 5, 18]. Previous studies reported that the production of hBD2 and SLPI by epithelial cells was induced by various microbes, microbial compounds, and cytokines [18–22]. Moreover, levels of cytokine and chemokine production were increased in response to different microorganisms and other cytokines [4, 5, 12, 18]. It is not known, however, if the expression of these innate immune mediators can be up-regulated by herbal plant extracts.

*Houttuynia cordata* Thunb (*H. cordata*), a herbal plant found in Asian countries, has long been in medical and dietary use by local people in this region [23]. It has antimicrobial activity against several bacteria and viruses [23–26], and also possesses other activities including anti-allergic [27], anti-inflammatory [28], antioxidative [29], and anticancer effects [30, 31]. Most laboratory studies have focused on the modulation of antimicrobial peptides and innate immune mediators by microbial components [18–21], but the role of herbal plant extracts on mucosal innate immunity is not well established. Therefore, the goal of this study was to evaluate the effects of *H. cordata* extract on the expression of hBD2 and SLPI as well as various cytokines and chemokines produced by human VECs. We demonstrated that *H. cordata* water extract induced the expression of hBD2 and SLPI mRNA in human VEC culture without measurable cytotoxicity. The secretion of hBD2 protein and the levels of IL-2 and IL-6 in the cell culture supernatants were also significantly increased in the presence of the extract.

## Methods

### Compound and reagent

Pure powdered extract of *H. cordata* was ordered from Erica Botanical Products (Xi’an, China). It was certified as free of bacterial or fungal contamination. The stock solution was prepared in sterile water. Tumor necrosis factor-alpha (TNF-α) used as a positive control for the induction of hBD2 and SLPI was ordered from PeproTech (Rocky Hill, CT, USA).

### Fibroblast cell culture

The fibroblast 3T3-J2 cells were kindly provided by Cary A. Moody from the Department of Microbiology and Immunology, University of North Carolina-Chapel Hill. The cells were cultured in Dulbecco’s Modified Eagles Medium (DMEM) (Life Technologies, Grand Island, NY, USA) with 10 % heat inactivated fetal bovine serum, L-glutamine, and penicillin-streptomycin at 37 °C in a humidified atmosphere containing 5 % CO_2_. For use as feeders for the vaginal epithelial cells, the fibroblasts were grown to 70–80 % confluence and irradiated with 6000 Rads.

### Primary human vaginal epithelial cell isolation and culture

Tissues routinely discarded from vaginal repair surgeries were harvested from three otherwise healthy adult women, with the understanding and written consent of each subject and according to ethical principles. Tissue harvesting and experimental procedures were approved by the Institutional Review Boards of the University of Washington and the Fred Hutchinson Cancer Research Center. Tissues were placed in ice-cooled calcium- and magnesium-free phosphate-buffered saline containing 100 U/ml penicillin, 100 μg/ml streptomycin and 2.5 μg/ml Fungizone (Life Technologies), and transported to the laboratory within 1 h of removal from the donor. The deep submucosa was removed with surgical scissors and the remaining vaginal mucosa was cut into 5 × 5 mm pieces, which were incubated at 4 °C for 18 h in 5 ml of a 25 U/ml dispase solution (BD Biosciences, Franklin Lakes, NJ, USA). The epithelial sheets were dissected off under a stereoscope and incubated for 10–12 min at 37 °C in 2 ml 0.05 % trypsin while gently shaking. The dispersed cells were poured through a 100 μm cell strainer into a 50 ml tube, pelleted by centrifugation, and resuspended in F medium (3:1 [v/v] F12 [Ham]-DMEM [Life Technologies], 5 % fetal bovine serum [Gemini Bio-Products, West Sacramento, CA, USA], 0.4 μg/ml hydrocortisone [Sigma, St. Louis, MO, USA], 5 μg/ml insulin [Gemini Bio-Products], 8.4 ng/ml cholera toxin [EMD Millipore, Billerica, MA, USA], 10 ng/ml epidermal growth factor [Life Technologies], 24 μg/ml adenine [Sigma], 100 U/ml penicillin, 100 μg/ml streptomycin [Life Technologies], and 2 mM glutamine [Life Technologies]). Primary VECs were plated into culture flasks in the presence of ~12,500/cm^2^ irradiated (6000 Rads) 3T3-J2 feeder fibroblasts and 10 μM of Rho kinase inhibitor Y27632 (Enzo Life Sciences, Farmingdale, NY, USA) was added [32, 33]. VECs were fed every 2–3 days and passaged when around 80 % confluence by 1 min treatment with 10 ml versene (Life Technologies) to remove the feeder cells, followed by 5 min treatment with trypsin/EDTA (Life Technologies). Dislodged VECs were washed and re-plated at ~2500 cells/cm^2^ with irradiated 3T3-J2 feeder fibroblasts.

### Cytotoxicity assay

To examine the toxicity of *H. cordata* water extract on primary human VECs, cells were evaluated using the CellTiter-Blue Cell Viability Assay (Promega, Madison, WI, USA). VECs were treated with 25–200 μg/ml *H. cordata* water extract for 18 h. After exposure, 20 μl of CellTiter-Blue Reagent was added to each well and the cells were cultured for 2 h. Subsequently, fluorescence in each well was measured with excitation/emission at 560/590 nm. Experiments were performed in triplicate and set up in three different donors. Viability of the cells was expressed as percent of the untreated negative control group.

### Stimulation of VECs by ***H. cordata*** water extract

Third passage VECs were treated with 25–200 μg/ml *H. cordata* water extract for 6 or 18 h. Untreated cells and cells treated with TNF-α were used as negative and positive controls, respectively. After treatment, total RNA was isolated from the cells for subsequent PCR assays, and culture supernatants were frozen at −80 °C for enzyme-linked immunosorbent assay (ELISA) and Luminex assay. Experiments were set up in three different donors; each experiment was performed in duplicate.

### RNA isolation, cDNA preparation and quantitative real-time reverse transcription PCR

Total RNA was isolated from VECs with the RNeasy Mini Kit and purified of contaminating DNA using RNase-free DNase (QIAGEN, Valencia, CA, USA). 1 μg of total RNA was reverse transcribed to complementary DNA (cDNA) using the iScript cDNA Synthesis Kit (Bio-Rad, Hercules, CA, USA). Quantitative real-time PCR was conducted using TaqMan Gene Expression Master Mix (Life Technologies) according to the manufacturer’s directions. The primers and probes for hBD2 and SLPI were as follows: hBD2 forward primer: 5′-TCCTGGTGAAGCTCCCA-3′; hBD2 reverse primer: 5′-CGCCTATACCACCAAAAACAC-3′; hBD2 probe: 5′-/56-FAM/AGGAGATAC/ZEN/AAGACCCTCATGGCTGA/3IABkFQ/-3′; SLPI forward primer: 5′-CAAGCGTGACTTGAAGTGTTG-3′; SLPI reverse primer: 5′-GAAAGGACCTGGACCACAC-3′; SLPI probe: 5′-/56-FAM/CCCTGTGAA/ZEN/AGCTTGATTCCTGCCA/3IABkFQ/-3′. Primers and probes for the housekeeping gene glyceraldehyde 3-phosphate dehydrogenase (GAPDH) were ordered from Life Technologies. Amplification conditions were initial denaturation at 96 °C for 15 min followed by 40 cycles of denaturation at 95 °C for 15 s and annealing/extension at 60 °C for 60 s. Amplification of each sample was performed in duplicate and normalized to the housekeeping gene. The relative expression was calculated by Pfaffl’s method [34] and expressed as the relative fold change of the untreated control group.

### ELISA and Luminex assay

Culture supernatants (collected as described above) were evaluated for secreted hBD2 and SLPI protein by ELISA (hBD2, Alpha Diagnostic, San Antonio, TX, USA; SLPI, R&D Systems, Minneapolis, MN, USA). The levels of interleukin (IL)-1β, IL-2, IL-4, IL-6, IL-8, IL-10, CC chemokine ligand (CCL) 2, CCL5, Interferon-gamma (IFN-γ), and TNF-α were measured by Magnetic Luminex Performance Assay (R&D Systems) according to the manufacturer’s instructions. Standard curves were generated in every set of experiments.

### Statistical analysis

Statistics were calculated with SPSS Statistics. One-way analysis of variance (ANOVA) and/or Kruskal-Wallis test was applied for analysis of differences between untreated cells and cells treated with *H. cordata*. *P*-values less than 0.05 were considered to indicate significantly different outcomes.

## Results

### Cytotoxicity of ***H. cordata*** water extract on primary VECs

**Fig. 1:**
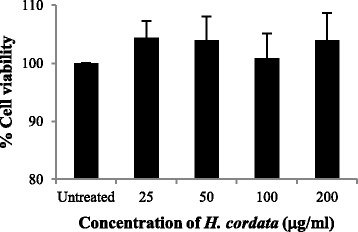
Cytotoxicity of VECs in the presence of *H. cordata* water extract. VECs were treated with *H. cordata* water extract for 18 h. Cell viability was evaluated by CellTiter-Blue cell viability assay. Results for triplicate experiments with three different donors were normalized to untreated cells, and mean and standard deviations (*error bars*) are shown

To test for cytotoxicity, primary VECs were treated with 25–200 μg/ml of *H. cordata* water extract for 18 h. After treatment, cell viability was measured by the CellTiter-Blue assay. VEC viability after treatment with *H. cordata* water extract was close to 100 % compared to untreated control cells (Fig. 1).

### Effect of ***H. cordata*** water extract on hBD2 and SLPI

**Fig. 2:**
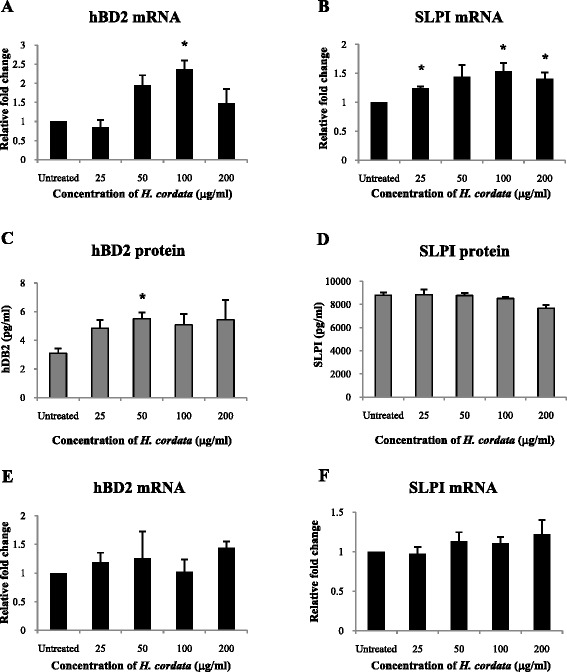
Effect of treatment with *H. cordata* on hBD2 and SLPI mRNA, and protein levels. VECs were treated with *H. cordata* water extract for 6 or 18 h. After treatment, the expression of hBD2 and SLPI mRNA was assessed by quantitative real-time RT-PCR. The expression of hBD2 and SLPI mRNA after treatment for 18 h (**a**, **b**) and 6 h (**e**, **f**) are shown as relative fold change compared to untreated control cells. Secretion of hBD2 and SLPI protein in VEC culture supernatants by ELISA, and mean ± SD values of secreted protein after treatment for 18 h are shown (**c**, **d**). In all graphs, results for duplicate experiments with three different donors for 18 h treatment and two different donors for 6 h treatment were normalized to untreated cells, and mean and standard deviations (*error bars*) are shown. *, *P* < 0.05 compared to untreated cells

Primary VECs were treated with 25–200 μg/ml of *H. cordata* water extract for 18 h. By using quantitative real-time reverse transcription PCR, the expression of hBD2 mRNA in VECs was found to be significantly increased by 2.37 fold after treatment with 100 μg/ml *H. cordata* (*p* < 0.05) compared to untreated cells (Fig. 2a). The expression of hBD2 mRNA after treatment with 50 or 200 μg/ml *H. cordata* was also increased, but not statistically significant (Fig. 2a). The expression of SLPI mRNA in VECs was significantly induced up to 1.53 fold after treatment with 25, 100, or 200 μg/ml *H. cordata* water extract (*P* < 0.05; Fig. 2b). SLPI mRNA was also increased with 50 μg/ml *H. cordata* but not statistically significant (Fig. 2b).

At the level of protein secretion, hBD2 was significantly increased from 3.1 pg/ml to 5.5 pg/ml after treatment with 50 μg/ml *H. cordata* water extract (*P* < 0.05) compared to untreated controls (Fig. 2c). The secretion of hBD2 by VECs after treatment with 25, 100 or 200 μg/ml *H. cordata* water extract was also increased, but not statistically significant (Fig. 2c). In contrast, the levels of secreted SLPI protein remained unchanged after treatment with low doses of *H. cordata*, and were slightly decreased with higher doses of the extract (Fig. 2d).

We also measured the expression of hBD2 and SLPI mRNA in VECs after being treated shortly with *H. cordata* water extract for up to 6 h. Unlike the case of 18 h treatment, 6 h of exposure with the extract had only slight effects on hBD2 or SLPI mRNA expression in VECs (Fig. 2e, f).

### Cytokine and chemokine secretion by VECs in the presence of ***H. cordata***

**Fig. 3:**
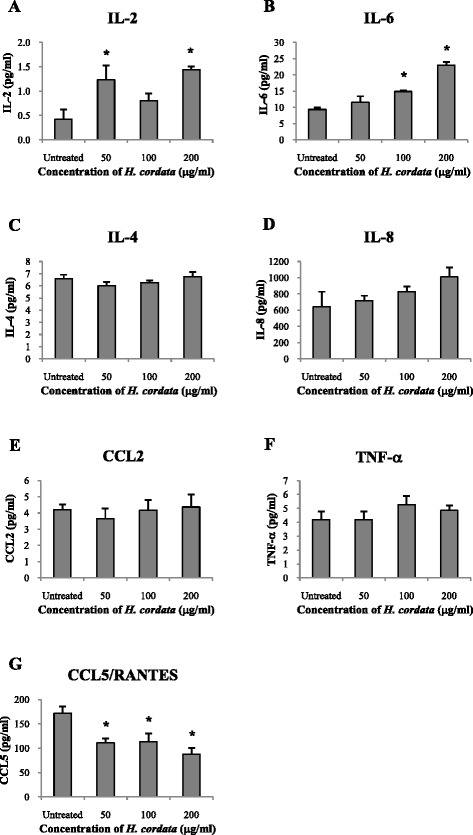
The secretion of cytokines and chemokines by VECs. VECs were treated with *H. cordata* water extract for 18 h. The levels of secreted cytokines and chemokines in cell culture supernatants were determined by Luminex assay. Mean and standard deviations (*error bars*) of cytokine concentrations after treatment are shown. Experiments were performed in duplicate with two different donors. *, *P* < 0.05 compared to untreated control group

Next, we measured the secretion of cytokines and chemokines in the cell culture supernatants after the cells were treated with 50, 100 or 200 μg/ml of *H. cordata* water extract for 18 h using Luminex assay. We found that the levels of secreted IL-2 in VEC culture supernatants were significantly higher (1.2-1.4 pg/ml) than untreated control cells (0.4 pg/ml) after treatment with 50 or 200, but not 100, μg/ml *H. cordata* water extract (*P* < 0.05; Fig. 3a). Similarly, the levels of secreted IL-6 were significantly increased (from 9.4 pg/ml to 15–23 pg/ml) after the cells were treated with 100 or 200 μg/ml *H. cordata* water extract (*P* < 0.05; Fig. 3b). However, levels of IL-4, IL-8, CCL2, and TNF-α were not statistically significant differences in the presence of the extract compared to untreated control cells (Fig. 3c, d, e, f). In contrast, the level of CCL5 was decreased from a basal level of 172 pg/ml to 88–114 pg/ml at the concentrations tested (*P* < 0.05; Fig. 3g). The levels of secreted IL-1β, IL-10, and IFN-γ proteins were also measured, however, they were at undetectable levels.

## Discussion

This study demonstrated that vaginal innate immune mediators are modulated by *H. cordata* water extract at the concentration up to 200 μg/ml without cytotoxicity. Increased hBD2 and SLPI gene expression, as well as hBD2, IL-2 and IL-6 protein secretion were found after the cells were treated with the extract for 18 h. In contrast, decreased CCL5 protein secretion by primary human VECs was noted.

Epithelial cells lining vagina provide the first line of defense against pathogenic microorganisms in the female genital tract. The cells play an active role both in physical and chemical barriers, including the production of antimicrobial peptides [1, 2, 6]. Our findings on the effects of *H. cordata* on modulating the innate immune mediators produced by VECs are consistent with that of a previous study by Luo et al. (2008), which reported that *H. cordata* can induce the expression of hBD2 in pulmonary epithelial cells [35].

Several studies revealed that hBD2 is an important antimicrobial peptide, because it is expressed in epithelial cells in response to microbial compounds, microbes and cytokines [18–21] and can inhibit several pathogenic bacteria [9], and *Candida* species [8]. Interestingly, it can inhibit HIV-1 transmission by directly inactivating HIV-1 virions, down-modulating HIV-1 co-receptor CXCR4, and inhibiting HIV replication [7, 15]. Thus, the induced expression of hBD2 by *H. cordata* water extract may be a useful feature of an anti-HIV microbicide or an antimicrobial agent for sexually transmitted diseases of the female reproductive tract.

Although the present study demonstrated that hBD2 was induced by the extract, it is not known whether the induction of antimicrobial peptides by the extract is sufficient to prevent or inhibit infection at the vaginal mucosa. Therefore, further study should be performed using functional assays to examine if the changes in antimicrobial peptide levels induced by the extract could prevent the proliferation of pathogens such as HIV and *C. albicans*. In addition, topical effects of the extract should be further explored *in vivo*.

SLPI is constitutively produced by several cell types including VECs [4, 36] and can also be induced by inflammatory mediators [22]. We found that the water extract of *H. cordata* increased the expression of SLPI mRNA by VECs after treatment for 18 h. However, the secretion of SLPI protein was not found to be increased. This discrepancy may be due to a stimulation time that was not suitable to detect SLPI protein secretion. It may also due to degradation of SLPI in VEC culture media as a recent study reported that the secreted protein level of SLPI did not reflect mRNA expression, suggesting rapid degradation of SLPI protein in the culture supernatant [37]. Further studies should be performed to determine the expression of SLPI in VECs after longer incubation of *H. cordata* to measure the level of SLPI in the late time point and to investigate the intracellular level of SLPI protein in VECs by a western blot.

Mucosal epithelial cells secrete cytokines and chemokines in response to microorganisms and other cytokines [5, 12]. Here, we demonstrated that the water extract of *H. cordata* increased the secretion of cytokines IL-2 and IL-6 by VECs. Although IL-2 is mainly produced by T lymphocytes, it can also be secreted at low levels by epithelial cells [38]. IL-6 is produced by different epithelia, including VECs [5, 12]. These cytokines play important roles in the host immune responses in both innate and adaptive immunity. IL-2 mediates the proliferation and activation of T cells and also activates natural killer cells and B-cells [13]. A previous study reported that highly active antiretroviral therapy combined with IL-2 can provide beneficial effects in the treatment of early HIV-1 infection [39]. IL-6 is a multifunctional cytokine with both pro-inflammatory and immunoregulatory functions [12, 13, 40]. It mediates the differentiation of B-cells and enhances immunoglobulin secretion [12, 13]. Moreover, IL-6 shows anti-inflammatory activity by inhibiting IL-1 and TNF [13]. On the other hand, IL-6 also has pro-inflammatory activity which may lead to undesirable effects such as inflammation. Further studies should be performed to elucidate if *H. cordata* extract might impose any adversary effects on the vaginal immunity. Of interest, previous studies demonstrated that IL-6 can inhibit HIV-1 replication in macrophages and plays a protective role in host immune responses against infections with various pathogens such as *Escherichia coli*, *Chlamydia trachomatis*, and *C. albicans* [41–44]. Thus, further studies should be carried out to explore whether cytokine alterations in VECs caused by *H. cordata* extract have functional effects, and whether the shift of cytokine profile in the culture supernatants can modify lymphocyte function and proliferation.

In addition to induction of IL-2 and IL-6, the water extract of *H. cordata* reduced the levels of CCL5 in the supernatants while the levels of IL-4 and TNF-α did not change. CCL5/RANTES has been identified as the HIV-suppressive factor [45], however, the decreased level of CCL5 after treatment with *H. cordata* may interrupt the effect of *H. cordata* in vaginal innate immunity regarding HIV-1 inhibition. Hence, the effect of *H. cordata* on HIV-1 inhibition in the target cells should be investigated in the future.

A recent study reported that the polysaccharide HCP-2 isolated from *H. cordata* water extract induced the secretion of IL-1β, TNF-α, and CCL5/RANTES by human peripheral blood mononuclear cells [46]. The production of IL-1β, IL-6, and TNF-α by mouse peritoneal macrophages was also enhanced by *H. cordata* [47]. Moreover, the production of IL-2 and IL-10 by mouse splenic lymphocytes was increased after treatment with *H. cordata*, whereas the level of IFN-γ decreased and the level of IL-4 remained unchanged [48]. These divergent results are likely due to varying methodological conditions, for example, tissue model, cell type, time of treatment, and dose of the extract.

The crude extract of *H. cordata* used in this study contains various substances. It is therefore unclear whether the observed modulation of immune mediators was caused by a single component or several. Thus, purification of different components of the crude extract of *H. cordata* should be done in the future. In addition, further studies should be performed to determine whether combinations of those active ingredients would be useful in the modulation of different immune mediators.

It has been shown that *H. cordata* possesses antimicrobial activities against several pathogenic microbes including *Staphylococcus aureus* and *E.* coli [23], however, the effects of *H. cordata* on the vaginal normal flora such as *Lactobacilli* are still not known and should also be determined.

## Conclusions

Our study indicates that *H. cordata* water extract induces the expression of antimicrobial peptides hBD2 and SLPI as well as the secretion of cytokines IL-2 and IL-6 by VECs and reduces the secretion of CCL5. These immune mediators have a significant role in innate and adaptive immune responses. These findings suggest that *H. cordata* extracts may be a useful immunomodulatory addition to vaginal microbicides and should be further investigated.

## Abbreviations

CCL: CC chemokine ligand
ELISA: Enzyme-linked immunosorbent assay
hBD2: Human β-defensin 2
*H. cordata*: *Houttuynia cordata* Thunb
HIV-1: Human immunodeficiency virus type 1
IL: Interleukin
IFN-γ: Interferon-gamma
SLPI: Secretory leukocyte protease inhibitor
TNF-α: Tumor necrosis factor-alpha
VECs: Vaginal epithelial cells
